# Barriers and Facilitators of Digital Transformation in Health Care: Mixed Methods Study

**DOI:** 10.2196/83551

**Published:** 2026-02-04

**Authors:** Marina Veldanova, Polina Glazkova, Elizaveta Krasilnikova, Marina Kazanfarova, Marina Bezuglova, Ekaterina Sosunova, Marina Zhuravleva

**Affiliations:** 1 SKOLKOVO School of Management Odintsovo Russian Federation; 2 Ipsos Comcon LLC Moscow Russian Federation

**Keywords:** digital transformation, physician barriers, technology acceptance, health care innovation, digital health, telemedicine, remote patient monitoring, clinical decision support systems, eHealth, mHealth

## Abstract

**Background:**

Digital transformation is now a fundamental component of health care systems worldwide. To develop effective digital health strategies, it is essential to examine physicians’ perspectives on the barriers and facilitators of implementation, with particular attention to regional and cultural factors influencing technology adoption.

**Objective:**

This study aims to identify and analyze key barriers and facilitators to the implementation of digital health technologies from physicians’ perspectives in Russia.

**Methods:**

A 2-phase nationwide mixed methods study was conducted involving 460 physicians from various specialties. The first phase comprised in-depth interviews with 10 physicians to develop a specialized questionnaire. The second phase involved a nationwide cross-sectional survey with 450 physicians using the developed questionnaire. Inclusion criteria were working in a Russian city with a population of more than 100,000, age 22 years and older, at least 3 years of specialty experience, and employment in public or private health care institutions. The analysis focused on 4 categories of digital health technologies: remote consultations, remote monitoring, digital diagnostic solutions, and clinical decision support systems.

**Results:**

The main barriers identified were fear of making erroneous decisions (25% of physicians), technical difficulties (up to 25%), and legal insecurity (21% of physicians). Notably, the barrier profile varied depending on the type of technology. Key drivers for implementation included time saving (59% of physicians), practical benefits (55% of physicians), and legal security (54% of physicians). Additionally, a convenient training organization was a crucial motivator, with the availability of free training (53% of physicians) and provision of study leave (52% of physicians). These facilitators were consistent across all categories of digital solutions. Based on these findings, key recommendations for the implementation of digital transformation in medical organizations were formulated.

**Conclusions:**

The findings highlight the need for comprehensive, technology-specific digital implementation strategies to improve health care digital transformation effectiveness, considering physician concerns about decision-making accuracy, technical challenges, and legal frameworks.

## Introduction

Digital transformation has become an integral part of modern health care systems around the world [1]. Technologies, such as telemedicine, remote patient monitoring, artificial intelligence–based diagnostics, and clinical decision support systems (CDSS), are increasingly seen as essential tools to address current and future challenges in health care [2]. The COVID-19 pandemic, in particular, has accelerated the adoption of certain digital solutions in health care, demonstrating their potential to support care continuity and mitigate public health crises [3,4]. Back in 2020, the World Health Organization approved the development of the Global Strategy on Digital Health 2020-2025 at the 73rd World Health Assembly [5].

The willingness of health care professionals, especially physicians, to accept new technologies and actively use them is a determining factor in the successful integration of digital solutions in health care [1]. Physicians play a key role in the implementation of digital solutions, influencing both their use and acceptance of digitalization by patients [6]. Therefore, understanding the specific barriers to implementation and factors that facilitate it, from a physician’s standpoint, is crucial for developing effective strategies for the implementation of digital solutions in health care [7]. At the same time, regional and cultural characteristics can have a critical impact on the typology of barriers and motivators in using various digital technologies.

This study aims to investigate the main barriers faced by physicians in using various digital technologies and to identify key drivers of health care digitalization in Russia.

## Methods

### Study Design

A 2-phase nationwide mixed methods study was conducted involving 460 physicians from various specialties.

### First Stage

At the first stage, in-depth online interviews (up to 1.5 hours) were conducted with 10 Moscow physicians with experience in using digital technologies. Among interview participants were 8 outpatient and polyclinic physicians and 2 inpatient physicians; 8 respondents represented the public sector, and 2 represented private clinics.

The analysis of the interviews allowed identifying key factors that facilitate and hinder digital transformation in health care. Based on the data obtained, a new questionnaire was developed to assess the attitude of physicians to digital transformation in health care and their experience of using digital technologies (Digital Health Readiness and Barriers Questionnaire for Physicians).

### Second Stage

At the second stage, an observational all-Russian study was conducted with 450 physicians using the questionnaire developed at the first stage.

To be included in the study, a physician had to meet the following criteria:

1. Work in a Russian city with a population of more than 100,000 people.
2. Age 22 years and older.
3. Work experience in the specialty for at least 3 years.
4. Work in public or private institutions (physicians working in departmental medical institutions were not allowed to participate).

To ensure an even and representative distribution of respondents, quotas were established for medical specialty and city of residence.

All respondents completed the online questionnaire developed in the first stage of the study. Completion of the questionnaire was voluntary and was processed anonymously and depersonalized.

This study analyzes the barriers to digital transformation in health care. The block includes 2 questions. The first one is devoted to the most significant obstacles to the implementation of digital technologies in practice. The physician is given 22 answer options; the respondent can mark up to 5 most relevant options. The full text of the question is provided in Multimedia Appendix 1.

For the ease of analysis, 22 statements were allocated into 5 domains—motivational, ability-related, process-related, physical (environmental factors), and social—reflecting the Motivation, Ability, Processing, Physical, and Social (MAPPS) framework grounded in behavioral theory. A comprehensive rationale and detailed description of each barrier group are provided in Multimedia Appendix 2.

The second question aimed to identify key factors that help overcome barriers to the implementation of digital technologies. Physicians were offered a list of 19 statements reflecting various advantages of using new digital solutions. Respondents assessed how likely it is that they would start using or use the relevant technologies more actively if the specified benefits were realized, using a 7-point scale: from 1 (definitely would not use or use more actively) to 7 (definitely would use or use more actively). The full text of the second question is provided in Multimedia Appendix 3.

The survey analyzed 4 categories of digital technologies (Table 1), with respondents separately noting the main barriers to the implementation of the relevant solutions for each category. This approach made it possible to identify the specifics and frequency of barriers depending on the type of digital technology, as well as to assess which barriers are most significant in each area of digital transformation in health care.

**Table 1:** Categories of digital technologies.

Abbreviated name	Full wording used in the survey
Remote physician-patient or physician-physician consultations	Remote (telemedicine) physician: patient consultations using audio or video communication Remote (telemedicine) physician: physician consultations using audio or video communication (eg, for emergency cases, scheduled consultations, online consultations)
Remote patient monitoring	Remote patient monitoring (eg, using medical sensors or an app to transmit one’s readings remotely to the physician via an app)
Technologies for diagnostics	Technologies for diagnostics (eg, computer vision to recognize X-rays, computed tomography scans, magnetic resonance imaging, and moles)
Clinical decision support systems	Systems to support physicians in making medical decisions (analysis of patient medical records, anamnesis, symptoms, results). For example, Webiomed, TOP-3, Sapia, and Onqueta.

The survey was conducted online from February 24 to March 17, 2025. The sample frame was created by randomly sending invitation links to all physicians registered on the Ipsos Comcon platform “Healthcare Professionals.” Emails containing a link to the survey were sent to 12,629 physicians; 1120 opened the link and viewed at least the first page and 450 physicians completed the survey. The survey response rate was 3.6%. Respondent recruitment was conducted using a quota sampling approach. A detailed description of the survey methodology, prepared in accordance with the CHERRIES (Checklist for Reporting Results of Internet E-Surveys), is provided in Multimedia Appendix 4.

### Statistical Data Processing

Descriptive statistics of the analyzed group are presented as percentages for qualitative variables. For quantitative variables, mean values and SDs were calculated. The study data were weighted according to official statistics on the distribution of primary care physicians and specialists in Moscow, St. Petersburg, and other cities [8]. Percentage calculations and data processing were performed using IBM SPSS Statistics (version 27).

### Ethical Considerations

#### Ethical Approval and Informed Consent

This study was approved by the Independent Ethics Committee of the Federal State Budget Scientific Institution “N.A. Semashko National Research Institute of Public Health” (protocol number 7, 2025). Written informed consent was obtained from all interview participants prior to conducting and audio recording the interviews. The study information materials provided comprehensive details regarding the research objectives, participant selection criteria, study procedures, time requirements, potential risks and benefits, participant rights and responsibilities, and data protection measures. Online survey respondents provided their consent by selecting the “Start” button following review of the introductory page, which contained information about survey content, estimated completion time, anonymity provisions, confidentiality protections, and research objectives. All participants were informed of their right to refuse participation or discontinue involvement in the research at any point without penalty. Informed consent was secured from all study participants. Participants in the online survey were offered a monetary incentive as compensation for their time and participation. Specifically, respondents were offered an electronic certificate worth 500 Russian rubles (US $6.44) for use at online hypermarkets. Interview participants did not receive monetary compensation.

#### Privacy and Confidentiality Protection

All survey responses were collected using anonymous data collection methods. Interview audio files and written transcripts underwent encryption protocols. Encrypted data access keys were maintained in a secure, password-protected local database with restricted access limited to MB, ES, and MZ only.

## Results

### Demographic Characteristics of the Sample

The survey on digital transformation in health care covered 450 physicians from 8 federal districts of Russia. Table 2 provides the characteristics of the study cohort of physicians.

The objective of the study was to obtain a result that would be representative of the digital transformation of physicians in Russia. It is obvious that the situation in large cities may differ from the results of the study in towns. According to official statistics, the share of physicians from Moscow and St. Petersburg (the 2 largest cities in the country) is 19% of all physicians in the Russian Federation [8]. The share of respondents practicing in Moscow and St. Petersburg was 28% (128/450) of the total sample, which indicates an insufficient representation of physicians from other regions and possible sample bias. To correct for this imbalance and ensure the representativeness of the data obtained, we applied poststratification weighting using official statistics on the regional distribution of physicians. The data below are given taking into account the weighting for the distribution of physicians by locality.

**Table 2:** Clinical and demographic characteristics of doctors.

Characteristics			Values (N=450)	
**Gender, n (%)**				
	Men		57 (12.7)	
	Women		393 (87.3)	
Age (years), mean (SD; range)			41.2 (9.57; 26-76)	
**Age (years), n (%)**				
	Up to 30		53 (11.8)	
	31-40		184 (40.9)	
	40-50		129 (28.7)	
	50+		84 (18.7)	
**Region of residence, n (%)**				
	Central Federal District		153 (34)	
	Northwestern Federal District		46 (10.2)	
	Southern Federal District		41 (9.1)	
	North Caucasian Federal District		4 (0.9)	
	Volga Federal District		115 (25.6)	
	Ural Federal District		30 (6.7)	
	Siberian Federal District		55 (12.2)	
	Far Eastern Federal District		6 (1.3)	
**City of residence, n (%)**				
	Moscow and St Petersburg		128 (28.4)	
	Other regions		322 (71.6)	
**Specialty, n (%)**				
	General practitioner or physician		110 (24.4)	
	Endocrinologist		71 (15.8)	
	Pediatrician		56 (12.4)	
	Gynecologist		45 (10)	
	Cardiologist		43 (9.6)	
	Neurologist		30 (6.7)	
	ENT^a^		17 (3.8)	
	Gastroenterologist		18 (4)	
	Surgeon		18 (4)	
	Pulmonologist		13 (2.9)	
	Ophthalmologist		8 (1.8)	
	Allergist		9 (2)	
	Urologist		9 (2)	
	Oncologist		1 (0.2)	
	Anesthesiologist-resuscitator		1 (0.2)	
	Functional diagnostics doctor		1 (0.2)	
Average length of service (years), mean (SD; range)			15.6 (8.96; 3-45)	
**Scientific degree, n (%)**				
	None		422 (93.8)	
	Candidate of Sciences		26 (5.8)	
	Doctor of Sciences		2 (0.4)	
**Type of institution, n (%)**				
	State		345 (76.7)	
	Municipal	228 (50.7)		
	Regional	94 (20.9)		
	Federal	23 (5.1)		
	Private		105 (23.3)	
**Type of admission, n (%)**				
	Outpatient		422 (93.8)	
	Inpatient		28 (6.2)	

^a^ENT: ear, nose, and throat.

### Key Barriers to Using Digital Technologies

Table 3 presents data on the frequency of various barriers that physicians encounter when implementing 4 digital technologies: remote consultations, remote patient monitoring, diagnostic technologies, and CDSS. The barriers were classified into 5 main groups: motivational, capability-related, process-related, physical, and social (MAPPS model). This classification was developed by Ipsos Comcon based on the behaviorist approach. For a detailed description and theoretical justification of barrier groups, see Multimedia Appendix 2.

For understanding the original distribution of responses, Multimedia Appendix 5 presents statistics corresponding to Table 3 based on the initial unweighted data, without adjustment for physician distribution. The results demonstrate that each type of technology is accompanied by a unique profile of barriers. For ease of perception, Table 4 shows the top 5 main barriers that physicians identified for each technology.

**Table 3:** Identification of barriers to the implementation of digital technologies.

Barriers^a,b^			Remote consultation (%)		Remote monitoring (%)		Technologies for diagnostics (%)		Systems to support physicians in making medical decisions (%)
**Motivation barriers^c^**			42		39.2		37.2		45.7
	I don’t see any practical benefit from using this technology in my daily work.	5.3		5.5		5.7		5.1	
	I am concerned about data privacy issues when using this technology.	20.9		15.7		9.3		15.3	
	I am concerned about the problem of excessive control over my work when using this technology.	9.4		5.2		4.3		9.8	
	This technology reduces the importance of physician’s work.	8.4		6.3		7.6		9.7	
	I don't trust the quality of this technology.	4.6		6		6.5		10.7	
	I am concerned about overdiagnosis when using this technology.	7.6		9.8		15.8		11.9	
**Capability-related barriers^c^**			28.7		38.9		48.5		48
	I don't have time to master this technology.	5.8		8.5		7.5		6.1	
	This technology is too complex to master.	1.4		2.5		4.2		5	
	I have no knowledge of specific products within this technology that could be used in my practice.	12.4		17.5		24.7		26	
	I don't have access to training courses to master this technology.	13.2		16.8		20.8		19.9	
	The technology requires personal investments to master it.	5.2		5.7		5.1		4.7	
**Process-related barriers^c^**			49.7		53.7		40.8		39.8
	I am not sure that this technology will work stably without delays and breakdowns.	25.3		29.2		18.9		18.2	
	I am afraid of making wrong decisions when using this technology.	22.2		26.4		26.8		22	
	Technology takes time without making work easier.	13.5		12.9		3.2		9	
**Environmental (physical) barriers^c^**			44.4		39.4		45.4		47.5
	I do not have technical base to master this technology (suitable equipment, software, communications).	20.5		24		31.6		26.7	
	Existing regulations do not include this technology or need to be revised.	20.5		13.4		12.5		15.3	
	This technology does not have qualified technical support.	14.8		11.3		10.4		13.3	
**Social barriers^c^**			47.5		39.3		33.9		40.4
	Management of my health care facility is not interested in using this technology.	13.9		15.1		14.3		20.7	
	My environment condemns the use of this technology.	1.1		0.9		0.6		1.5	
	I prefer to use other long-proven methods rather than this technology.	8.6		5.8		9.8		5.9	
	I feel a lack of legal security when using this technology.	29.8		21		14.9		17.5	
	None of the above.	14.2		13		13.6		11.1	

^a^The data provided are weighted by the distribution of physicians across populated areas of the Russian Federation. Multimedia Appendix 5 provides unweighted data.

^b^The table shows the percentage of doctors who selected each answer. Each respondent could select up to 5 answers.

^c^The total for each category shows the percentage of doctors who selected at least one of the category barriers.

**Table 4:** The top 5 main barriers to different types of digital technologies in health care.

Barriers to using digital technologies^a^		Values (%)
**Barriers to remote consultations (top 5)**		
	Lack of legal protection	29.8
	Doubts about stable operation of technology	25.3
	Fears of making wrong decisions	22.2
	Data privacy concerns	20.9
	Lack of technical base	20.5
**Barriers to remote monitoring (top 5)**		
	Doubts about stable operation of technology	29.2
	Fear of making wrong decisions	26.4
	Lack of technical base	24
	Lack of legal security	21
	Lack of knowledge about products	17.5
**Barriers to technologies for diagnostics (top 5)**		
	Lack of technical base	31.6
	Fear of making wrong decisions	26.8
	Lack of knowledge about products	24.7
	Lack of training courses	20.8
	Fear of overdiagnosis	15.8
**Barriers to systems to support physicians in making medical decisions (top 5)**		
	Lack of technical base	26.7
	Lack of knowledge about products	26
	Lack of interest from management	20.7
	Lack of training courses	19.9
	Lack of legal protection	17.5

^a^The data provided are weighted by the distribution of physicians across populated areas of the Russian Federation.

### Remote Physician-Patient or Physician-Physician Consultations

The most common barriers for remote consultations are social barriers (47.5%) and process-related barriers (49.7%). The most frequently mentioned barriers include lack of legal protection (29.8%, the highest rate among all technologies), doubts about the stability of the technology (25.3%), and fears of making wrong decisions (22.2%). In addition, 20.9% of doctors expressed concerns about data privacy, which is the highest rate for this barrier among all technologies. Motivational barriers were encountered by 42% of doctors. It is the second most frequent value among all groups.

Thus, despite their relative prevalence and technical accessibility, remote consultations are often perceived by physicians as legally and organizationally vulnerable, especially in conditions of insufficient regulatory support and lack of confidence in data protection.

### Remote Monitoring

For remote patient monitoring technologies, process-related barriers were dominant (53.7%), which was the highest value among all barrier categories. The most frequently noted barriers were system instability (29.2%) and concerns about decision-making errors (26.4%).

There was also a high proportion of physicians who indicated a lack of technical expertise to master this technology (24%), a feeling of legal insecurity (21%), and a lack of knowledge about specific products (17.5%).

Thus, physicians face challenges in integrating remote monitoring technologies into daily practice due to a wide range of concerns, but technical and methodological barriers related to reliability, safety, and the need for new professional skills come to the fore.

### Technologies for Diagnostics

As barriers to implementation of diagnostic technologies, physicians most often indicated insufficient technical base (31.6%) and fear of making wrong decisions (26.8%) when using technology.

Technologies for diagnostics provided the highest values in terms of opportunity-related barriers (48.5%), particularly lack of product knowledge (24.7%) and lack of access to training resources (20.8%).

Also, 15.8% of respondents expressed fear of overdiagnosis**,** which is the highest among all technologies.

These results indicate that digital diagnostic solutions are perceived by physicians as technologically complex and not transparent enough, requiring serious support for implementation and methodological adaptation.

### CDSS

CDSS turned out to be the least acceptable for respondents in terms of motivational barriers (45.7%) and environmental barriers (47.5%).

For this group of technologies, the most common problems were lack of technical base (26.7%), lack of knowledge about products (26%), and unavailability of training courses (19.9%).

Also, 20.7% of doctors indicated a lack of support from management of the health care facility (which is the highest indicator of this barrier among all technologies). This result demonstrates the importance of active participation and the initiative of management in integrating digital solutions into clinical practice.

An important issue for physicians remains legal security (17.5%) when using this group of technologies.

Physicians also noted a lack of trust in the quality of technology (10.7%), a feeling of excessive control over their professional activities (9.8%), and a decrease in the significance of the role of physicians (9.7%). Although these motivational barriers were selected by a relatively small number of respondents, they were most frequently identified for CDSS compared to other types of digital technologies, which characterizes the low level of trust of doctors in this type of technology.

Thus, CDSS is perceived by physicians as a problematic technology both in terms of technical infrastructure, organizational support, and professional trust.

Overall, the most common barriers to implementation of all digital solutions are technical and organizational difficulties, fear of making wrong decisions, and a sense of legal insecurity (Table 4). At the same time, the intensity of the expression of individual barriers varies depending on the type of technology: for example, for remote consultations, legal and regulatory barriers come to the fore, while for the other 3 types of digital technologies, technical difficulties play a key role. This emphasizes the need for differentiated implementation support strategies that take into account the specifics of each type of digital tool.

**Figure 1:**
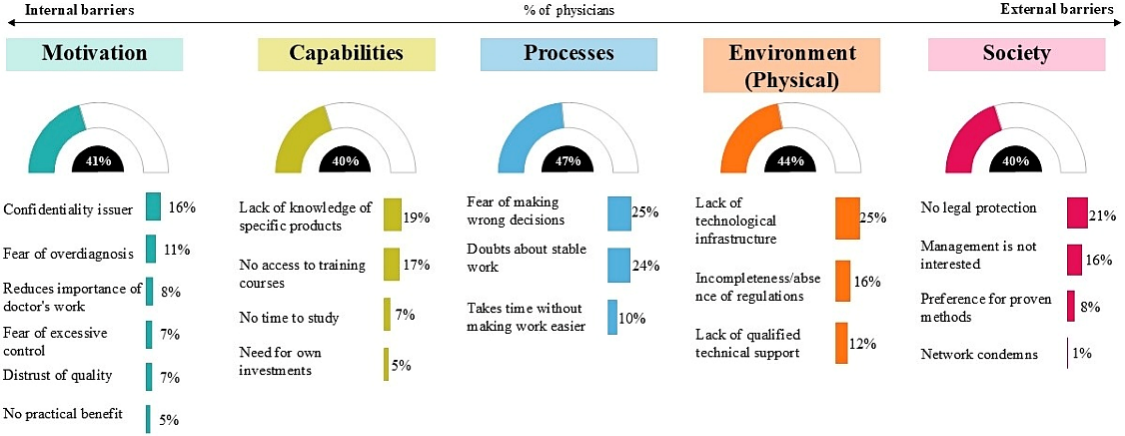
General distribution of barriers to the implementation of digital technologies in health care from the point of view of doctors. The graph shows the percentage of physicians who selected a particular answer option. For category data, the percentage of physicians who selected at least one of the category barriers is shown. The data presented are weighted by the distribution of doctors across populated areas of the Russian Federation.

Figure 1 shows a generalized distribution of barriers that physicians face when implementing digital technologies in clinical practice (the total result for all types of technologies).

Although the distribution of barriers was generally fairly even (from 40% to 47%), procedural difficulties came to the fore, noted by 47% of physicians. Most often, they indicated fear of making wrong decisions (25%) and doubts about the stable operation of digital systems (24%).

Among environmental barriers (44%), the leading one is the lack of technical base (25%), and among social barriers (40%), the first place is taken by the lack of legal security (21%).

A significant proportion are also capability-related barriers (40%), primarily a lack of knowledge about specific products (19%). Likewise, motivational barriers (41%) reflect physicians’ internal doubts: primarily concerns about data privacy (16%).

Thus, the figure illustrates that barriers to implementation of digital technologies in health care are multifaceted and cover both the internal attitudes of doctors and external organizational and legal restrictions, which require comprehensive solutions at the level of the health care system.

### Drivers of Digital Technologies Implementation in Health Care

Table 5 shows the top 5 main drivers that, in the opinion of physicians, can help overcome barriers to implementation of digital technologies (Multimedia Appendix 6 provides a complete table of the distribution of drivers for different types of technologies).

Notably, while the barrier profile differed depending on the type of digital technology, the leading drivers were similar across all technology types.

Time-saving potential was consistently ranked first, with 56% to 62% of votes. Also, practical benefits were in the top 3 drivers for all technology types. This highlights that physicians are primarily interested in real functional efficiency and time savings in a busy practice environment.

For all types of technologies, legal security was included in the top 5 main criteria necessary for using the technology. This criterion was most significant for remote consultations (57.7%) and remote monitoring (54.6%).

**Table 5:** The top 5 main factors contributing to the introduction of digital technologies in health care, according to physicians.

Drivers of digital technologies implementation in health care^a,b^		Values (%)
**Drivers to remote consultations (top 5)**		
	Technology will save time	62.2
	Technology will deliver practical benefits in daily work	58.3
	Legal protection when using this technology	57.7
	Free or at the expense of the health care institution	55.8
	Management will allow taking study leave	54.4
**Drivers to remote monitoring (top 5)**		
	Technology will save time	58.1
	Legal protection when using this technology	54.6
	Technology will deliver practical benefits	54
	Free or at the expense of the health care institution	53.2
	Management will allow taking study leave	51.1
**Drivers to technologies for diagnostics (top 5)**		
	Technology will save time	56
	Free or at the expense of the health care institution	52.8
	Technology will deliver practical benefits	52.2
	Management will allow taking study leave	51.7
	Legal protection when using this technology	51.4
**Drivers to systems to support physicians in making medical decisions (top 5)**		
	Technology will save time	58.3
	Technology will deliver practical benefits	54.5
	Legal protection when using this technology	51.2
	Free or at the expense of the health care institution	50.3
	Interface will be accessible and understandable	49.4

^a^The data provided are weighted by the distribution of physicians across populated areas of the Russian Federation.

^b^Based on the answers to the question: “How likely is it that you would start using/more actively use the following technologies when implementing the ideas on a scale from 1 to 7?” The table shows the percentage of respondents who chose 7 points for this answer (a physician would definitely start using digital technology if the conditions specified in the statement were met).

**Figure 2:**
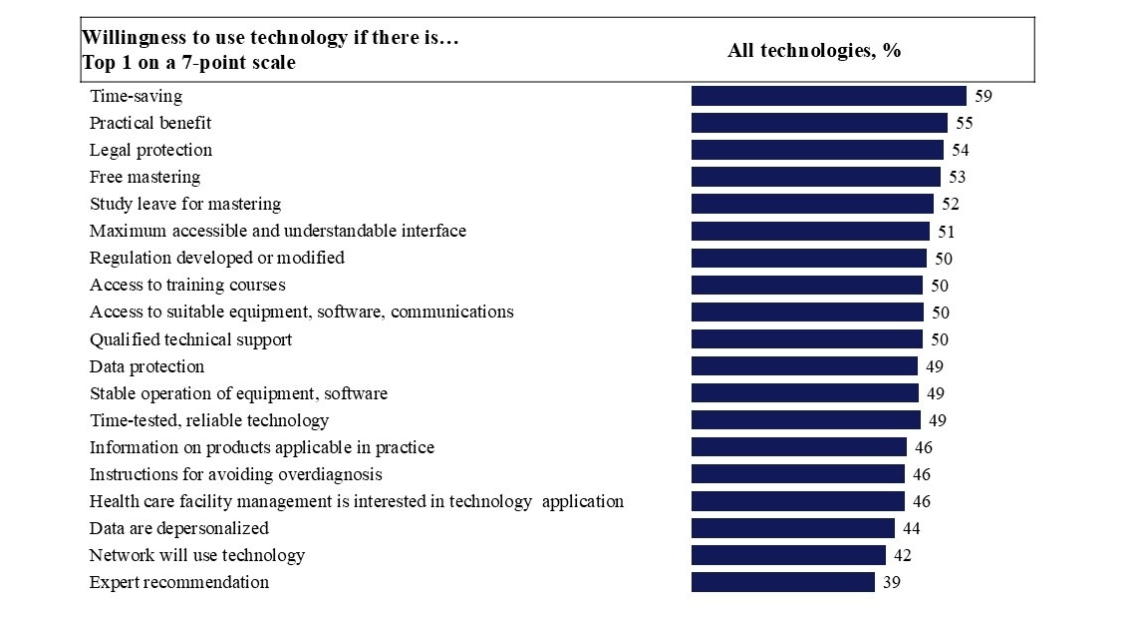
Key factors contributing to the implementation of digital technologies, according to physicians. The data presented are weighted by the distribution of doctors across populated areas of the Russian Federation. Based on the answer to the question: “How likely are you to start using or more actively use the following technologies when implementing ideas on a scale from 1 to 7? The figure shows the percentage of respondents who chose 7 points for this answer (a physician would definitely start using digital technology if the conditions specified in the statement were met).

Another important block of incentives is related to the reduction of barriers to learning and the technical ease of using the technology: availability of free training, study leave, and a simple interface were also included in the top 5 factors. This indicates the need not only to implement technologies, but also to create a supportive learning environment, especially in conditions of time constraints for medical personnel. All drivers for different technologies are summarized in Figure 2.

The results highlight that physicians perceive digitalization primarily through the prism of daily efficiency, legal security, and organizational support.

The main driver for the implementation of all digital technologies is saving doctors’ time. This is important to consider when implementing digital technologies in health care institutions.

## Discussion

### Barriers to Implementation of Digital Technologies in Health Care

In recent years, digitalization in health care has become an integral part of medical practice. However, the introduction of digital technologies is accompanied by a number of barriers that must be taken into account for the successful integration of innovations into clinical practice.

The study of 450 physicians in Russia found that key barriers to the implementation of digital technologies include technical difficulties, fear of making wrong medical decisions, and concerns about legal insecurity.

Lack of technical infrastructure (25%) and doubts about the stability of technology (24%) were the key barriers to the implementation of digital technologies. These data are relevant to that from the international studies, in particular, according to the largest umbrella meta-analysis of 108 systematic reviews [1], infrastructural and technical barriers to implementation of digital technologies in health care rank first in frequency worldwide.

Along with technical difficulties, fear of making wrong decisions (25%) based on the use of digital technologies was ranked first in this study. A number of studies also demonstrate that it is the fear of physicians to make a mistake when relying on digital tools that is an important barrier to the implementation of digital technologies. Thus, a survey of 1449 physicians by the American College of Physicians, conducted in 2019, showed that 29% of specialists see the risk of potential medical errors as one of the main obstacles to the implementation of telemedicine [9,10]. Another study showed that 42.1% of American doctors are concerned about a decrease in the quality of care provided when using telemedicine [11]. It is worth noting that this fear has certain grounds. Thus, in a study of medical malpractice cases related to the use of remote telephone consultations, the most common accusation was incorrect diagnosis (68%), and the most common form of damage was death (44%) [12]. Systematic review by Kim et al [13] found that IT issues in health care can significantly disrupt care processes and lead to errors in clinical decision-making, delays in treatment, and even harm to patients. In 53% of the included studies, IT-related issues were associated with actual or potential harm to patients, including deaths.

Thus, in addition to technical difficulties, it is the fear of physicians to make mistakes due to inaccuracies in digital systems that remains the key barrier to digitalization in health care. It can be overcome by improving the validation of algorithms, ensuring transparency of systems, and clear legal regulation in digital technology use.

The feeling of legal insecurity when using digital technologies, identified by 24% of Russian physicians, reflects one of the most significant and persistent problems in digital transformation in health care. This barrier manifests itself in the concerns of medical workers about the possible legal consequences of errors associated with the use of digital solutions, as well as in the unclear distribution of responsibility between a physician, an institution, and a technology developer [14,15].

Legal and regulatory risks are one of the main factors hindering the implementation of digital solutions in clinical practice [16]. In particular, it is noted that the existing legal framework is often not adapted to the specifics of digital technologies: there are no clear standards for medical data storage, transfer, and processing, as well as for determining liability in the event of errors or incidents related to digital tools [9,14,16]. This leads to the fact that physicians are forced to rely on general norms of professional duty and ethics, which increases uncertainty and reduces the willingness to use innovations [14].

Thus, the feeling of legal insecurity is not only a subjective fear, but also an objectively determined barrier associated with the insufficient development of the regulatory framework, absence of clear standards and mechanisms for distributing responsibility. To overcome this obstacle, it is necessary to develop and implement modern legal and ethical standards adapted to digital reality, as well as professional medical communities, to actively participate in the formation of regulatory policy [9,14,16].

Another important conclusion from the study is that a significant portion of physicians (16%) identified the lack of management support in the implementation of digital technologies as a key barrier. Specifically, for the CDSS, this factor was among the top 3 barriers and was noted by 20.7% of physicians. Organizational support and management interest are often considered important factors in the successful implementation of digital technologies in health care [17,18]. This study emphasizes the need for strategic management involvement and the development of leadership competencies for the successful implementation of digital technologies.

A literature review revealed that a significant number of studies have identified concerns among health care professionals that implementation of digital technologies will increase their workload, which is a significant barrier to digital decision-making [1,19-21]. This fear appears paradoxical, since many digital technologies are initially developed to optimize workflows, save time, and improve the efficiency of clinical decision-making [22-24]. However, this phenomenon requires serious attention from researchers and practitioners, since health care professionals’ perception of a potential increase in workload remains a significant barrier to digital transformation.

In this study, the fear of losing time was also mentioned by physicians, although it did not come to the forefront. Only 10% of physicians identified “takes time and does not make work easier” barrier, and only 7% of physicians were not ready to spend time mastering the technology. It is also worth noting that according to this study, only 7% of physicians identified distrust of digital technologies as a barrier, and 5% a low level of practical benefit. This may indicate a high psychological readiness of Russian medical workers for digital transformation. Such differences emphasize the importance of taking into account national and cultural contexts when developing strategies for implementing digital solutions in health care.

In general, the spread of barriers among the 5 MAPPS categories was fairly uniform, ranging from 40% to 47% of respondents per category (Figure 1). This distribution highlights the need for a comprehensive and multilevel approach to addressing various obstacles to successfully advancing digital transformation in health care.

A more detailed analysis revealed that process-related and environmental barriers received somewhat higher ratings, 47% and 44%, respectively. These included fears of making errors, doubts about the stability of digital systems, and a lack of technical infrastructure with qualified support. Meanwhile, motivation, capability-related, and social barriers were noted by slightly fewer respondents, approximately 40%-41%. This pattern suggests a relatively high level of motivation among Russian physicians and a general readiness within the medical community to adopt digital technologies. At the same time, it underscores the critical need to enhance technical infrastructure and ensure the stable operation of digital tools with professional technical support.

The barriers were unevenly distributed among different technology groups. For example, for telemedicine technologies, a notably high percentage of physicians (47.5%) reported encountering social barriers, primarily linked to perceived legal insecurity. In contrast, for systems to support physicians in making medical decisions, process-related barriers (39.8%) and social barriers (40.4%) were minimal, whereas motivation barriers (45.7%) and capability-related barriers (48%) predominated. This divergence reflects the specific perceptions and challenges associated with implementing different digital solutions in clinical practice and underscores the necessity for a differentiated approach to their support and regulation.

The study findings emphasize the need for a comprehensive and tailored approach to overcoming barriers. This approach should consider the specific type of technology to determine the most effective implementation strategies.

### Drivers of Digital Technologies Implementation in Health Care

In contrast to the diverse profile of barriers, the Russian study found remarkable consistency in the leading enablers of technology adoption across all 4 categories of digital technologies, pointing to universal motivators for physicians.

Physicians value digital solutions primarily for 2 specific advantages: time savings and real practical benefits. It is important for them that the technology makes work easier and speeds it up, rather than adding extra tasks [1,19,20]. If developers clearly show how much time the new system saves and how it fits into the routine process [25], physicians are willing to use it. Thus, it is important to demonstrate to physicians how the digital tool simplifies the routine and frees up time for the patient and other important matters.

Perceived legal security consistently ranked among the top 5 factors facilitating adoption for all types of technologies, being most significant for remote consultations (57.7%) and remote patient monitoring (54.6%). The lack of legal clarity is a significant barrier, and conversely, its presence acts as a powerful catalyst for the adoption of digital technologies. Physicians seek concrete assurances that they will not face undue professional or legal liability for potential errors, data breaches, or unintended consequences arising from the use of new, complex digital tools [9,14,16]. Thus, it is necessary not only to create a clear and transparent legal framework for the use of digital technologies in health care, but also to ensure that physicians are informed about the relevant legal norms and regulations in an accessible and understandable manner.

“Free learning/at the expense of the health care institution” and “management will allow taking study leave” were among the top 5 factors of assistance for all types of technologies. This underlines the readiness to learn and the importance of competent organizational support for this process.

Unfortunately, the introduction of new technologies often requires physicians to master new skills without interrupting their clinical practice. Thus, this only increases their workload during the period of mastering the technology. This explains why, in a number of studies, the key barrier to implementation of digital technologies was the fear of increasing physicians’ workload [1,19,20].

Our research shows that study leave and management-paid training make new technologies much more attractive to physicians.

Thus, the implementation of digital technologies in health care is a complex process of creating a supporting organizational ecosystem. Key factors in this process are the provision of technical infrastructure, legal transparency, training, and management support. The absence or weakness of any single component can undermine the entire digital transformation effort. True, sustainable transformation requires a coordinated, systemic approach in which all these elements are strategically aligned and continuously strengthened.

### Limitations

This study has several limitations that should be considered when interpreting the findings. First, the study inclusion criteria required physicians to work in large urban centers in Russia, which inherently limits the generalizability of results to health care providers practicing in smaller towns or rural areas. The experiences and barriers faced by physicians in less populated or resource-constrained settings may differ significantly from those in larger urban centers.

Second, the reliance on online questionnaires might have introduced a selection bias, as physicians without reliable access to the necessary technology or internet connectivity were unable to participate.

Third, data collection was based on self-reported questionnaires, which are subject to inherent biases, including social desirability bias. Respondents may have underreported negative attitudes or challenges due to perceived social or professional expectations.

Despite these limitations, the study provides valuable insights into physician perspectives on digital technology adoption in health care within the sampled population. Future research should aim to include a more diverse sample and consider mixed data collection methods to minimize bias and enhance generalizability.

It is important to note that the process of health care digitalization depends not only on physicians but also significantly on patient readiness and engagement. Therefore, studying patient-related factors is crucial and represents a key focus for our future research endeavors.

### Conclusions

The study showed that the key barriers to the introduction of digital technologies in health care in Russia are technical difficulties (lack of infrastructure, unstable operation of systems), fear of making wrong decisions based on digital data, and a feeling of legal insecurity.

Lack of knowledge about specific products, lack of management support, and limited training opportunities also play a significant role. The profile of barriers varies depending on the type of digital technology, which highlights the need for differentiated approaches to their implementation. At the same time, the leading drivers for physicians are time savings, practical benefits, legal protection, availability of free training, and organizational support. These motivators are universal for all categories of digital solutions and reflect doctors' desire to improve efficiency and reduce workload in a busy environment.

Based on the conducted research, we formulated key recommendations for the implementation of digital transformation in medical organizations.

1. Development of technical infrastructure: ensuring stable operation of digital systems, access to necessary equipment, and integration with existing work processes.
2. Improvement of the legal and regulatory framework: development of clear standards and mechanisms for sharing responsibility, and ensuring that physicians are clearly informed about current legal regulations.
3. Implementation of educational programmes: arranging free training and providing physicians with study leave to master new technologies, which will increase digital literacy and reduce resistance to change.
4. Strengthening of organizational support: involving management of medical institutions in digitalization processes, forming a culture of leadership and support for innovation.
5. Demonstration of practical value: demonstrating to clinicians how digital tools save time, simplify routine tasks, and improve the quality of care.
6. Considering specifics of technologies: developing implementation strategies taking into account the specifics of each category of digital solutions and the profile of relevant barriers.

The comprehensive implementation of these measures will increase the readiness of the medical community for digital transformation and ensure sustainable implementation of innovative solutions in health care.

## Appendix

Multimedia Appendix 1 Question 1. Different physicians mention different barriers to using digital technologies in healthcare. Are there any that are also relevant to you? Please select up to 5 answers. The physician is asked to answer a question on each of the four categories of digital technologies separately.

Multimedia Appendix 2 Justification for the division of barriers in accordance with the MAPPS model.

Multimedia Appendix 3 Question 2: A team of experts has already thought about some of the problems that arise when implementing digital technologies in the life of a physician. Now we will show you some ideas, please rate how likely it is that you would start using / use the following technologies more actively when implementing these ideas on a scale from 1 to 7, where 1 - definitely would not start using / use more actively, 7 - definitely would start using / use more actively. The physician is asked to answer a question on each of the four categories of digital technologies separately.

Multimedia Appendix 4 CHERRIES Checklist.

Multimedia Appendix 5 Identification of barriers to implementation of digital technologies.

Multimedia Appendix 6 Main drivers for overcoming barriers. The data are weighted by distribution of physicians by populated areas of the Russian Federation. The table shows the percentage of respondents who chose 7 points for this answer (physician would definitely start using digital technology if the conditions specified in the statement were met).

## Abbreviations

CDSS: clinical decision support system
CHERRIES: Checklist for Reporting Results of Internet E-Surveys
MAPPS: Motivation, Ability, Processing, Physical, and Social

## Data Availability

The datasets generated or analyzed during this study are not publicly available due to confidentiality and ethical restrictions protecting participant privacy, but are available from the corresponding author on reasonable request, in compliance with institutional and ethical guidelines.
