# Supplementary material for: Barriers and Facilitators of Digital Transformation in Health Care: Mixed Methods Study
Source: J Particip Med. 2026 Feb 4;18:e83551. doi: 10.2196/83551 (PMC12917481; doi:10.2196/83551)
Supplement: Multimedia Appendix 1 [file jopm_v18i1e83551_app1.docx]

Appendix A.1 Digital Health Readiness and Barriers Questionnaire for Physicians.

Section: Barriers to Digital Transformation in Healthcare

**Table A1.** Different physicians mention different barriers to using digital technologies in healthcare. Are there any that are also relevant to you? Please select up to 5 answers^a^.

| 1 | Motivation barriers | I don't see any practical benefit from using this technology in my daily work. |
| --- | --- | --- |
| 2 |  | I am concerned about data privacy issues when using this technology |
| 3 |  | I am concerned about the problem of excessive control over my work when using this technology. |
| 4 |  | This technology reduces the importance of physicians’ work |
| 5 |  | I don't trust the quality of this technology. |
| 6 |  | I am concerned about overdiagnosis when using this technology. |
| 7 | Capability-related barriers | I don't have time to master this technology. |
| 8 |  | This technology is too complex to master. |
| 9 |  | I have no knowledge of specific products within this technology that could be used in my practice. |
| 10 |  | I don't have access to training courses to master this technology. |
| 11 |  | The technology requires personal investments to master it |
| 12 | Process-related barriers | I am not sure that this technology will work stably without delays and breakdowns. |
| 13 |  | I am afraid of making wrong decisions when using this technology. |
| 14 |  | Technology takes time without making work easier |
| 15 | Environmental barriers | I do not have technical base to master this technology (suitable equipment, software, communications) |
| 16 |  | Existing regulations do not include this technology or need to be revised. |
| 17 |  | This technology does not have qualified technical support. |
| 18 | Social barriers | Management of my healthcare facility is not interested in using this technology. |
| 19 |  | My environment condemns the use of this technology |
| 20 |  | I prefer to use other long-proven methods rather than this technology. |
| 21 |  | I feel a lack of legal security when using this technology |
|  |  | None of the above |

^a^The physician is asked to answer a question on each of the four categories of digital technologies separately.
