# Supplementary material for: Barriers and Facilitators of Digital Transformation in Health Care: Mixed Methods Study
Source: J Particip Med. 2026 Feb 4;18:e83551. doi: 10.2196/83551 (PMC12917481; doi:10.2196/83551)
Supplement: Multimedia Appendix 2 [file jopm_v18i1e83551_app2.docx]

## Appendix B

**Table B.** Justification for the division of barriers in accordance with the MAPPS model

| **MAPPS DIMENSION** | **MAPPS CATEGORY** | **Theory** |
| --- | --- | --- |
| **Motivation** | Outcome expectations | Based on Festinger (1962) and Friston (2018) and Kruglanskia et al. (2018). A large body of evidence shows how beliefs about how an outcome will unfold have a significant impact on impacting behavior. |
|  | Emotion | Appraisal theory - works on the basis that we appraise our bodily associations on the basis of a number of criteria which determines the type of emotion. Based on Smith and Ellsworth (1985) and Scherer et al. (2013). |
|  | Internalization | Self Determination Theory – theory of the mechanisms needed to internalise motivation. Based on Ryan and Deci (2000). Censydiam “plugs in” here as a way to articulate various sources or needs driving internal motivation. |
|  | Identity | Americus Reed (2012) examines the role identity (consumers’ self-concepts) plays in guiding buying decisions |
|  | Self-efficacy | Based on social cognitive theory – theory of knowledge acquisition developed by Albert Bandura which explore knowledge acquisition |
| **Ability** | Capability | Also based on social cognitive theory but this aspect focuses on human capabilities of symbolising, self-regulation, self-reflection, and vicarious learning. This helps understand what contributes to the development of knowledge and abilities. |
|  | Routines | The framework developed by Hobson et al. (2018) that we draw upon provides a way of examining their function from a psychological perspective, thereby helping to underpin individual behaviour change. |
| Processing | Decision forces and Adaptive processing | A range of theories from the judgement and decision-making literature and informed by DDMM |
| Physical | Environmental factors | Meder and colleagues (2018) provide an overview of different types of environments to help better identity how suited they are for different types of interventions. |
| Social | Social norms | Based on Perkins and Berkowitz (1986) Cialdini et al. (1990) |
|  | Cultural norms | Based on Schwartz (1994) cultural values |
