# Supplementary material for: Barriers and Facilitators of Digital Transformation in Health Care: Mixed Methods Study
Source: J Particip Med. 2026 Feb 4;18:e83551. doi: 10.2196/83551 (PMC12917481; doi:10.2196/83551)
Supplement: Multimedia Appendix 3 [file jopm_v18i1e83551_app3.docx]

Appendix A.2 Digital Health Readiness and Barriers Questionnaire for Physicians.

Section: Barriers to Digital Transformation in Healthcare

**Table A2.** Question 2: A team of experts has already thought about some of the problems that arise when implementing digital technologies in the life of a physician. Now we will show you some ideas, please rate how likely it is that you would start using / use the following technologies more actively when implementing these ideas on a scale from 1 to 7, where 1 - definitely would not start using / use more actively, 7 - definitely would start using / use more actively.^a^

| 1 | The technology will bring practical benefits to your daily work. |
| --- | --- |
| 2 | Data will be reliably protected from leaks |
| 3 | Data in the system will be stored in a depersonalized manner |
| 4 | Experts in your professional environment will recommend the use of this technology. |
| 5 | The technology will be time-tested and reliable. |
| 6 | There will be clear instructions on how to avoid overdiagnosis |
| 7 | The management will allow you to take study leave to master this technology |
| 8 | The technology interface will be as accessible and understandable as possible. |
| 9 | You will be informed about specific products that are applicable to your professional practice. |
| 10 | You will have access to training courses to master this technology. |
| 11 | The development of the technology will be free of charge / will be carried out at the expense of the healthcare facility |
| 12 | The technology developer will guarantee stable operation of equipment and software |
| 13 | Technology will save your time |
| 14 | You will have access to suitable equipment, software, communications |
| 15 | Regulatory and legal acts will be created or revised for the use of this technology. |
| 16 | The technology will be followed by qualified technical support |
| 17 | The management of the health care institution will be interested in using this technology. |
| 18 | Your environment will also use this technology. |
| 19 | You will be legally protected when using this technology. |

^a^The physician is asked to answer a question on each of the four categories of digital technologies separately.
