# Supplementary material for: Barriers and Facilitators of Digital Transformation in Health Care: Mixed Methods Study
Source: J Particip Med. 2026 Feb 4;18:e83551. doi: 10.2196/83551 (PMC12917481; doi:10.2196/83551)
Supplement: Multimedia Appendix 4 [file jopm_v18i1e83551_app4.docx]

**Appendix C**

**Table C. Checklist for Reporting Results of Internet E-Surveys (CHERRIES)**

| ***Checklist Item*** | ***Explanation*** |
| --- | --- |
| Describe survey design | The target population for this study consisted of primary care physicians practicing in Russian cities with populations over 100,000. The sample frame was created by randomly sending invitation links to all physicians registered on the Ipsos Comcon platform "Healthcare Professionals". Respondent recruitment was conducted using quota sampling approach. |
| IRB approval | Study has been approved by independent ethics committee FSSBI «N.A. Semashko National Research Institute of Public Health», protocol №7, 2025. |
| Informed consent | Online survey respondents provided their consent by selecting the "Start" button following review of the introductory page, which contained information about survey content, estimated completion time, anonymity provisions, confidentiality protections, and research objectives. All participants were informed of their right to refuse participation or discontinue involvement in the research at any point without penalty. Informed consent was secured from all study participants. |
| Data protection | To protect unauthorized access to personal data, the following mechanisms are implemented:  1. Authentication  Verifies user identity using credentials (e.g., username/password).  Enhanced with multi-factor authentication (MFA) via authenticator app.  2. Authorization  Ensures users only access data necessary for their role.  3. Auditing and Monitoring  Log all access attempts and data operations.  Enable real-time alerts for suspicious behavior (e.g., multiple failed logins, unusual data exports).  4. Data Minimization & Anonymization  Collect only necessary data.  Anonymize or pseudonymize data where possible (e.g., replacing names with IDs).  Reduces risk if data is compromised.  5. Compliance with Regulations  Adherence to the The Russian Federal Law on Personal Data (No. 152-FZ)  6. Physical Security  Secure server rooms with surveillance and access logs.  Prevent unauthorized physical access to devices.  7. Regular Security Training & Incident Response  Employees trained to recognize phishing and social engineering.  Incident response plans in place for data breaches. |
| Development and testing | The usability and technical functionality of the electronic questionnaire as well as its logic and adherence to the paper questionnaire had been tested before the fieldwork. |
| Open survey versus closed survey | It was a password-protected survey |
| Contact mode | The initial contact with potential participants was established by sending invitation letters to the participants' e-mail |
| Advertising the survey | The survey was not advertised. Potential participants received invitation letters announcing the survey via email. |
| Web/E-mail | The link to the survey questionnaire was sent out through e-mail. The questionnaire itself was posted on a web site. Automatic method for capturing responses has been used. |
| Context | The web site has been created for the survey specifically and contained survey questions only. It was available only by the link and could not be found trough a search engine |
| Mandatory/voluntary | Participation in the survey was voluntary for all respondents. |
| Incentives | As an incentive for participation, respondents were offered a certificate worth 500 rubles for an online hypermarket. |
| Time/Date | The survey was conducted online from February 24 to March 17, 2025. |
| Randomization of items or questionnaires | To minimize the order effect, the response options were rotated. |
| Adaptive questioning | Yes, adaptive questioning was used. Questions regarding barriers and drivers for different types of technologies were not shown to respondents who answered 'I don’t know anything about this' on the preliminary awareness question about the specific technology. Respondents with other answers were presented with the main questions about barriers and drivers. |
| Number of Items | Questions have been shown on separate screens. The longest question contained 22 response options with multiple choice. |
| Number of screens (pages) | The paper questionnaire had 17 pages. Electronic questionnaire shown to the respondents had 47 screens. This study analyzes only one section «Barriers to Digital Transformation in Healthcare». This section consisted of two questions presented on two separate screens. The questions were asked separately for each type of technology. |
| Completeness check | Consistency or completeness checks have been implemented for each question using Dimensions soft (here is the demo-link). A respondent could not move on to the next question if the answer was contradictory or incomplete. Non-response options have been shown where it was applicable. |
| Review step | Respondents were not able to review or change their answers. |
| Unique site visitor | The survey links sent via emails were unique for each doctor. At the end of the survey, the doctors provided their names and phone numbers, and these answers were compared with the mailing list. |
| View rate (Ratio of unique survey visitors/unique site visitors) | The rate is 100% as the web-site was available only by a link sent to respondents via email. The emails were sent to 12 629 doctors, 1120 opened the link and saw at least the first page. |
| Participation rate (Ratio of unique visitors who agreed to participate/unique first survey page visitors) | The “recruitment” rate is 91% (1022 doctors filled in the first survey page out of 1120) |
| Completion rate (Ratio of users who finished the survey/users who agreed to participate) | 44%: 450 completes out of 1022 |
| Cookies used | Duplicate entries avoided by preventing users access to the survey twice. Cookies were not used. |
| IP check | IP addresses of the client computer were not used to identify potential duplicate entries from the same user. |
| Log file analysis | The links had a unique ID for each doctor and could be completed only once. In case of duplicate entry “You have already participated in the survey” text was shown. |
| Registration | The survey was closed. Users had to login to complete the questionnaire. The survey never displayed a second time once the user had filled it in |
| Handling of incomplete questionnaires | Only completed questionnaires were analyzed |
| Questionnaires submitted with an atypical timestamp | 10 minutes cut-off was used. It was determined based on questionnaire testing results. Median completion time was about 31 minutes. The time to complete the section « Barriers to Digital Transformation in Healthcare »﻿ accounted for approximately 20% of the total time. |
| Statistical correction | Weighting by city size and doctors’ specialty type (primary care or not) was used to adjust for the non-representative sample |
