# Supplementary material for: Barriers and Facilitators of Digital Transformation in Health Care: Mixed Methods Study
Source: J Particip Med. 2026 Feb 4;18:e83551. doi: 10.2196/83551 (PMC12917481; doi:10.2196/83551)
Supplement: Multimedia Appendix 5 [file jopm_v18i1e83551_app5.docx]

**Table S1.**

| **Barriers**^a^ | | **Remote consultation, %** | **Remote monitoring, %** | **Technologies**  **for diagnostics, %** | **Systems to support physicians in making medical decisions, %** |
| --- | --- | --- | --- | --- | --- |
| **Motivation barriers** | | 42.3 | 39.6 | 37.5 | 46.1 |
|  | I don't see any practical benefit from using this technology in my daily work. | 5.6 | 5.5 | 5.7 | 5.6 |
|  | I am concerned about data privacy issues when using this technology | 20.9 | 16.6 | 9.3 | 14.5 |
|  | I am concerned about the problem of excessive control over my work when using this technology. | 9.7 | 5.2 | 4.6 | 10 |
|  | This technology reduces the importance of physician's work | 8.8 | 5.9 | 7.7 | 9.7 |
|  | I don't trust the quality of this technology. | 4.5 | 5.5 | 6.4 | 11.5 |
|  | I am concerned about overdiagnosis when using this technology. | 7.4 | 10 | 15.9 | 11.9 |
| **Capability-related barriers** | | 28.4 | 38.6 | 48.1 | 46.8 |
|  | I don't have time to master this technology. | 5.9 | 8.5 | 8 | 5.9 |
|  | This technology is too complex to master. | 1.4 | 2.6 | 4.6 | 4.8 |
|  | I have no knowledge of specific products within this technology that could be used in my practice. | 12.2 | 16.8 | 23.9 | 25.3 |
|  | I don't have access to training courses to master this technology. | 13.1 | 17.1 | 20.6 | 19.7 |
|  | The technology requires personal investments to master it | 5 | 5.5 | 4.9 | 4.5 |
| **Process-related barriers** | | 49.8 | 52.8 | 41.1 | 40.1 |
|  | I am not sure that this technology will work stably without delays and breakdowns. | 25.2 | 28.2 | 19 | 18.2 |
|  | I am afraid of making wrong decisions when using this technology. | 22.7 | 26.5 | 26.5 | 23 |
|  | Technology takes time without making work easier | 13.1 | 12.6 | 3.6 | 9.3 |
| **Environmental barriers** | | 43.9 | 40.3 | 45.8 | 47.2 |
|  | I do not have technical base to master this technology (suitable equipment, software, communications) | 19.6 | 24.6 | 31.9 | 25.7 |
|  | Existing regulations do not include this technology or need to be revised. | 20.9 | 13.7 | 12.6 | 15.6 |
|  | This technology does not have qualified technical support. | 14.6 | 11.1 | 10.3 | 13.8 |
| **Social barriers** | | 47.5 | 38.9 | 33.2 | 40.9 |
|  | Management of my healthcare facility is not interested in using this technology. | 14.2 | 14.9 | 13.9 | 20.4 |
|  | My environment condemns the use of this technology | 1.1 | 0.9 | 0.5 | 1.5 |
|  | I prefer to use other long-proven methods rather than this technology. | 8.3 | 5.7 | 9.5 | 5.9 |
|  | I feel a lack of legal security when using this technology | 30.2 | 20.6 | 14.7 | 18.2 |
|  | None of the above | 14 | 12.6 | 13.1 | 11.2 |

^a^ The table shows the percentage of doctors who selected each answer. Each respondent could select up to 5 answers. The total for each category (highlighted in color) shows the % of doctors who selected at least one of the category barriers.
