# Supplementary material for: Barriers and Facilitators of Digital Transformation in Health Care: Mixed Methods Study
Source: J Particip Med. 2026 Feb 4;18:e83551. doi: 10.2196/83551 (PMC12917481; doi:10.2196/83551)
Supplement: Multimedia Appendix 6 [file jopm_v18i1e83551_app6.docx]

## Appendix C

**Table C.** Main drivers for overcoming barriers^a^.

| **Statement** | **Remote physician-patient or physician - physician consultations, %** | **Remote monitoring, %** | **Technologies for diagnostics, %** | **Systems to support physicians in making medical decisions, %** |
| --- | --- | --- | --- | --- |
| Technology will bring practical benefits to your daily work | 58.3 | 54 | 52.2 | 54.5 |
| Data will be reliably protected from leaks | 52.5 | 46.9 | 46.9 | 46.9 |
| Data in the system will be stored in a depersonalized manner | 43.9 | 42.8 | 42.5 | 45.5 |
| Experts in your professional environment will recommend the use of this technology | 37.9 | 38.4 | 40 | 42.4 |
| Technology will be time-tested and reliable | 52 | 49.6 | 46.6 | 46.7 |
| There will be clear instructions on how to avoid overdiagnosis | 46.9 | 44.4 | 45.4 | 45.4 |
| Management will allow for a study leave to master technology | 54.4 | 51.1 | 51.7 | 49.3 |
| Technology interface will be as accessible and understandable as possible | 53.6 | 50 | 50.1 | 49.4 |
| You will be informed about specific products that are applicable to your professional practice | 47.4 | 46.7 | 44.2 | 45.9 |
| You will have access to training courses to master technology | 51.8 | 47.2 | 49.9 | 49.2 |
| Development of technology will be free of charge / will be carried out at the expense of healthcare facility | 55.8 | 53.2 | 52.8 | 50.3 |
| Technology developer will guarantee stable operation of equipment and software | 48.9 | 48.7 | 49 | 48.3 |
| Technology will save your time | 62.2 | 58.1 | 56 | 58.3 |
| You will have access to suitable equipment, software, communications | 50.6 | 49.2 | 47.7 | 52.1 |
| Regulatory and legal acts will be developed or revised for the use of technology | 51.9 | 49.2 | 49.2 | 50.8 |
| Technology will have qualified technical support | 50.9 | 50.9 | 46.9 | 48.9 |
| Management of health care institution will be interested in using technology | 45.7 | 45.3 | 45.8 | 46.5 |
| Your environment will also use this technology | 43.7 | 38.8 | 40.6 | 43.9 |

^a^The data are weighted by distribution of physicians by populated areas of the Russian Federation. The table shows the percentage of respondents who chose 7 points for this answer (physician would definitely start using digital technology if the conditions specified in the statement were met).
